# Supplementary material for: Numerical simulation study on optimization of key technical parameters of unpowered dust removal system in a gas-solid two-phase flow field
Source: PLoS One. 2025 May 30;20(5):e0324379. doi: 10.1371/journal.pone.0324379 (PMC12124849; doi:10.1371/journal.pone.0324379)
Supplement: Attachment — Submitted filename: Response to the reviewers.docx [file pone.0324379.s002.docx]

**Response to the reviewers**

Ref: PONE-D-24-47959-Numerical Simulation Study on Optimization of Key Technical Parameters of Unpowered Dust Removal System in a Gas-solid Two-phase Flow Field

Dear Prof. Muhammad Shakaib,

Thank you very much for your letter and the comments from the referees about our paper submitted to ***Plos One.***

We have checked the manuscript and revised it according to the comments. We submit here the revised manuscript as well as a list of changes.

If you have any question about this paper, please don’t hesitate to let me know.

Sincerely yours,

Lv Chen

**Response to Editor：**

Thanks for your comments on our paper. We have revised our paper according to your comments:

(1) **The convergence criterion of 0.001 is high. The simulations need to be performed with lower values such as 0.00001 or 0.000001.**

**Reply：**According to the editor’s comment, We have refined the convergence criterion and conducted numerical simulations in accordance with the updated criterion.

**In article：**

**Table 1 Model parameter setting**

| **Parameter** | **Parameter setting** | **Parameter** | **Parameter setting** |
| --- | --- | --- | --- |
| Solver | Pressure basis solver | Entrance and exit pressure | 0 Pa |
| Turbulent flow model | Standard k-ε model | Turbulent intensity | 4% |
| Entrance boundary type | Pressure inlet | Solution method | SIMPLEC |
| Exit boundary type | Pressure outlet | Convergence criteria | 1e-06 |

(2) **The regulatory framework /industry standards for other regions should also be considered.**

**Reply：**According to the editor’s comment, we also incorporated dust exposure limit standards from other regions, including both European and American standards.

**In article：**

**Table 5 Individual long-term respiratory dust concentration (C_TWA_) test results**

| **Testing element** | **Job site** | **Detected results C_TWA_(mg/m^3^)** | | **China standard**  **(mg/m^3^)** | **European standard**  **（mg/m^3^）** | **American standard**  **（mg/m^3^）** |
| --- | --- | --- | --- | --- | --- | --- |
|  |  | **Before transformation** | **After transformation** |  |  |  |
| Raw coal unit | East coal pit | 3.48 | 0.31 | 4 | 3 | 1.8 |
|  | Eastern raw coal blending warehouse | **5.52** | 0.44 | 4 | 3 | 1.8 |
|  | Western coal pit | **5.12** | 0.39 | 4 | 3 | 1.8 |
|  | Western raw coal blending warehouse | 3.56 | 0.32 | 4 | 3 | 1.8 |
| Coal Washing unit | Under East washing building raw coal bunker | **6.6** | 0.52 | 4 | 3 | 1.8 |
|  | East washing building roof belt | 3.4 | 0.30 | 4 | 3 | 1.8 |
|  | Eastern clean coal bunker allocation warehouse | 3.88 | 0.36 | 4 | 3 | 1.8 |
|  | Western District raw coal bunker | **5.08** | 0.76 | 4 | 3 | 1.8 |
|  | On the western clean coal bunker | **6.42** | 1.07 | 4 | 3 | 1.8 |
| Coal conveying unit | West coal slime belt | 4.75 | 0.25 | 4 | 3 | 1.8 |

**Response to Reviewer 1:**

Thanks for your comments on our paper. We have revised our paper according to your comments:

(1) **The title of the manuscript carries the words "numerical simulation study" but the author do not mention any in-house code or commercial software package to conduct the CFD simulations.**

**Reply：**According to the reviewer’s comment, We have provided additional clarification in the paper. Specifically, we utilized a commercially available and widely recognized software to conduct numerical simulations of the engineering problems associated with the unpowered dust removal system discussed herein.

**In article：**

**3.1 Physical model and meshing**

The equal-proportion simplified physical model was established using SolidWorks modeling software, as illustrated in Fig. 3. Subsequently, this model was imported into Fluent numerical simulation software to perform the subsequent simulation calculations.

**Fig.3 Physical model**

……

(2) **The vast majority of the references are cited in the introduction. There are no references to support the numerical simulation methodology. The numerical results should be validated with respect to a peer-reviewed publication.**

**Reply：**According to the reviewer’s comment, In the introduction, we have incorporated a discussion on the numerical simulation method and its research advancements. However, existing literature offers limited numerical simulation results for this specific engineering problem, making it challenging to provide adequate data validation support. Consequently, our research group intends to construct a similar experimental simulation platform (as shown in Fig. 1) to verify the numerical simulation results and conduct further analysis and discussion.

**Fig.1 Test platform**

**In article：**

**0 Introduction**

**……**

Since the 1960s, numerous domestic and international scholars have initiated research on dust control technology. As awareness of the hazards associated with dust has increased, significant advancements have been made in this field. Various innovative dust removal equipment, technologies, and materials have been continuously introduced[1-4]. In 1986, American scholar Martin Engineering[5] developed Inertial Flow Technology (IFT). This technology focuses on designing a novel curved coal drop system. Controlling the trajectory of falling material to align closely with the inner wall of the coal drop system forms a stable material flow, thereby reducing collisions between the material and the inner wall and among material particles, thus minimizing dust generation. The design of the coal drop system ensures that the material's exit velocity approximates the belt speed, thereby reducing induced wind speed and suppressing dust dispersion. With the advancement of discrete element method (DEM) simulation software and the enhancement of computer computing power, Cleary, Nordell, et al. employed DEM simulations to model material transport trajectories under actual working conditions. Their results demonstrated that designing the coal drop system in the transport system as a curve can more effectively control the flow direction of discrete materials and reduce the final material outlet velocity, thereby minimizing material impact and dust generation[6]. Building on the original cyclone dust collector design, H. J. Luckner [7] introduced additional modifications to enhance its efficiency for power plants. This improvement aimed to address the low dust removal efficiency despite mechanical dust removal technology's simplicity and ease of maintenance. Test results showed that the modified design expanded the range of captured particle sizes and significantly improved the overall dust removal efficiency of the collector. Xiang Xu[8] combined plasma with a ring electrode through capacitive coupling to refine the original electrode design and voltage levels, thereby enhancing dust removal efficiency. Experimental findings indicated that while dust removal efficiency increased, the required dust removal time increased for larger dust particles. Heinz Antes[9] introduced circulating electrostatic dust removal technology into the mine belt transportation system to reduce dust diffusion in large spaces. Experimental results demonstrated that this technology can achieve a dust reduction efficiency of up to 90% in the workplace. However, it also involves high power consumption and significant manual maintenance requirements, increasing operational costs. Shi Jinhua et al.[10] also applied spray sprinkler technology to control dust in the belt coal conveying system, achieving satisfactory dust suppression effects. Due to climatic limitations, the dust removal equipment and pipelines often freeze during cold winters, causing water conveyance and spray systems to malfunction. Zhang Wenbin et al.[11] proposed using bag filter dust removal methods to reduce dust concentrations to meet relevant national standard. Nevertheless, this approach suffers from the short service life of the cloth bags, necessitating timely replacement after accumulating a certain amount of coal dust, thereby increasing the maintenance workload. Moreover, inadequate or damaged bag sealing can increase coal dust pollution. Some scholars[12] have suggested employing micro-power dust removal technology for dust control. However, this method also incurs high power consumption and requires substantial manual maintenance, contributing to elevated operational costs.

**……**

**Response to Reviewer 2:**

(1) **The text requires an overall revision regarding grammar and technical terms, so it should be edited specifically for the language by some native editors.**

**Reply：**According to the reviewer’s comment, We have revised the grammar and technical terms of the text.

(2) **In its present form, the abstract of the work does not sound well. The abstract should contain the following: objectives, methods/analysis, findings, and novelty/improvement.**

**Reply：**We appreciate the reviewers' guidance on abstract writing and have revised the abstract under your suggestions.

**In article：**

**Abstract：**Based on the engineering background of the Huaibei Coal Preparation Plant in Anhui Province, China, this study aims to effectively mitigate dust pollution during the blanking process at coal transfer points. Given the limitations of conventional dust control measures, a numerical simulation was conducted using the Euler model, soft sphere collision model, and discrete element method (DEM). The simulation incorporated the two-way coupling effect between gas-solid two-phase flow and the collision-adhesion dynamics among particles. We examined the migration behavior of dust within an unpowered dust removal system under various technical parameters. Results indicate that installing a return pipe significantly reduces dust overflow caused by impact airflow during blanking. Specifically, when the return pipe diameter is 500 mm, and the horizontal distance between the drainage port of the return pipe and the bottom of the blanking pipe is 2000 mm, the dust removal efficiency reaches its optimal level. Field tests confirmed that after implementing the improved dust removal system, the ambient air dust concentration decreased to less than 2 mg/m³, representing a reduction of approximately 92.02% compared to pre-transformation levels. This approach overcomes the limitations of traditional dust prevention technologies, inhibits dust generation at its source within the coal conveying system, effectively reduces working space dust concentrations, and ensures occupational health and safety for workers.

(3) **The literature review is superficial. There is only a list of papers without any discussion about them. The gap in knowledge should be clearly presented. Even the motivation of the study should be clearly highlighted. At the end of the introduction, introduce the novelty of your study.**

**Reply：**Following the expert's recommendations, we thoroughly revised the introduction section. When citing others' research findings, we provided a critical evaluation and discussed our perspective on these results, as well as identified common challenges encountered in this field of study. At the end of the introduction, we outlined this research's primary focus and innovative aspects.

**In article：**

**0 Introduction**

In the daily production process, the generation of various types of dust is inevitable. When the aerodynamic diameter of harmful dust particles is below 7.07 μm, these fine particulates can enter the human respiratory system through inhalation, leading to pneumoconiosis in workers over time. According to relevant statistics, there were over 975,000 cases of pneumoconiosis in 2019, including 873,000 cases of occupational pneumoconiosis, accounting for approximately 90% of all occupational diseases in China. Consequently, pneumoconiosis caused by excessive dust concentration has become the most severe occupational hazard in the country. In industries such as mining, power plants, and material terminals, belt conveyors are widely used due to their advantages of high capacity, efficient material transmission, and ease of installation and operation. However, during the material transfer process on belt conveyors, significant amounts of dust are generated when materials fall at each transfer point. Dust concentration measurements at operational sites indicate that dust levels at conveyor transfer points typically exceed safety standards, posing substantial risks to enterprise safety and employee health. This issue represents a critical technical challenge that production enterprises must urgently address.

Since the 1960s, numerous domestic and international scholars have initiated research on dust control technology. As awareness of the hazards associated with dust has increased, significant advancements have been made in this field. Various innovative dust removal equipment, technologies, and materials have been continuously introduced[1-4]. In 1986, American scholar Martin Engineering[5] developed Inertial Flow Technology (IFT). This technology focuses on designing a novel curved coal drop system. Controlling the trajectory of falling material to align closely with the inner wall of the coal drop system forms a stable material flow, thereby reducing collisions between the material and the inner wall and among material particles, thus minimizing dust generation. The design of the coal drop system ensures that the material's exit velocity approximates the belt speed, thereby reducing induced wind speed and suppressing dust dispersion. With the advancement of discrete element method (DEM) simulation software and the enhancement of computer computing power, Cleary, Nordell, et al. employed DEM simulations to model material transport trajectories under actual working conditions. Their results demonstrated that designing the coal drop system in the transport system as a curve can more effectively control the flow direction of discrete materials and reduce the final material outlet velocity, thereby minimizing material impact and dust generation[6]. Building on the original cyclone dust collector design, H. J. Luckner [7] introduced additional modifications to enhance its efficiency for power plants. This improvement aimed to address the low dust removal efficiency despite mechanical dust removal technology's simplicity and ease of maintenance. Test results showed that the modified design expanded the range of captured particle sizes and significantly improved the overall dust removal efficiency of the collector. Xiang Xu[8] combined plasma with a ring electrode through capacitive coupling to refine the original electrode design and voltage levels, thereby enhancing dust removal efficiency. Experimental findings indicated that while dust removal efficiency increased, the required dust removal time increased for larger dust particles. Heinz Antes[9] introduced circulating electrostatic dust removal technology into the mine belt transportation system to reduce dust diffusion in large spaces. Experimental results demonstrated that this technology can achieve a dust reduction efficiency of up to 90% in the workplace. However, it also involves high power consumption and significant manual maintenance requirements, increasing operational costs. Shi Jinhua et al.[10] also applied spray sprinkler technology to control dust in the belt coal conveying system, achieving satisfactory dust suppression effects. Due to climatic limitations, the dust removal equipment and pipelines often freeze during cold winters, causing water conveyance and spray systems to malfunction. Zhang Wenbin et al.[11] proposed using bag filter dust removal methods to reduce dust concentrations to meet relevant national standard. Nevertheless, this approach suffers from the short service life of the cloth bags, necessitating timely replacement after accumulating a certain amount of coal dust, thereby increasing the maintenance workload. Moreover, inadequate or damaged bag sealing can increase coal dust pollution. Some scholars[12] have suggested employing micro-power dust removal technology for dust control. However, this method also incurs high power consumption and requires substantial manual maintenance, contributing to elevated operational costs.

In summary, the current domestic and foreign scholars ' research on dust removal technology mainly focuses on the technical improvement of the original dust removal device and the comprehensive application of the existing dust removal technology. Given the characteristics of the working conditions of the transshipment point of the coal conveying system, aiming at the problem of excessive dust control at the material transshipment point, the author takes Huaibei Coal Preparation Plant in Anhui Province of China as the research object. Based on the dust generation mechanism analysis and migration law, the idea of applying the curved coal drop pipe combined with the unpowered dust removal device to comprehensively control the problem of excessive dust at the transshipment point is proposed.

(4) **The innovation of the paper is not completely clear, kindly mention the novelty of the present analysis.**

**Reply：**According to the reviewer’s comment, at the end of the introduction, we point out the innovative work of our research.

**In article：**

**0 Introduction**

**……**

In summary, the current domestic and foreign scholars ' research on dust removal technology mainly focuses on the technical improvement of the original dust removal device and the comprehensive application of the existing dust removal technology. Given the characteristics of the working conditions of the transshipment point of the coal conveying system, aiming at the problem of excessive dust control at the material transshipment point, the author takes Huaibei Coal Preparation Plant in Anhui Province of China as the research object. Based on the dust generation mechanism analysis and migration law, the idea of applying the curved coal drop pipe combined with the unpowered dust removal device to comprehensively control the problem of excessive dust at the transshipment point is proposed.

(5) **Aside from the aim stated in the title, the research gap and the goals of the research are not specified which leads to the reader missing the significance of the research.**

**Reply：**We appreciate the valuable suggestions provided by the review expert，We address the research gap by discussing the findings of previous studies. In the initial section of the introduction, we elucidate the study's objectives within the context of practical engineering applications. As for the significance of the research, we make a supplementary explanation in the last part of the introduction.

**In article：**

**0 Introduction**

In the daily production process, the generation of various types of dust is inevitable. When the aerodynamic diameter of harmful dust particles is below 7.07 μm, these fine particulates can enter the human respiratory system through inhalation, leading to pneumoconiosis in workers over time. According to relevant statistics, there were over 975,000 cases of pneumoconiosis in 2019, including 873,000 cases of occupational pneumoconiosis, accounting for approximately 90% of all occupational diseases in China. Consequently, pneumoconiosis caused by excessive dust concentration has become the most severe occupational hazard in the country. In industries such as mining, power plants, and material terminals, belt conveyors are widely used due to their advantages of high capacity, efficient material transmission, and ease of installation and operation. However, during the material transfer process on belt conveyors, significant amounts of dust are generated when materials fall at each transfer point. Dust concentration measurements at operational sites indicate that dust levels at conveyor transfer points typically exceed safety standards, posing substantial risks to enterprise safety and employee health. This issue represents a critical technical challenge that production enterprises must urgently address.

Since the 1960s, numerous domestic and international scholars have initiated research on dust control technology. As awareness of the hazards associated with dust has increased, significant advancements have been made in this field. Various innovative dust removal equipment, technologies, and materials have been continuously introduced[1-4]. In 1986, American scholar Martin Engineering[5] developed Inertial Flow Technology (IFT). This technology focuses on designing a novel curved coal drop system. Controlling the trajectory of falling material to align closely with the inner wall of the coal drop system forms a stable material flow, thereby reducing collisions between the material and the inner wall and among material particles, thus minimizing dust generation. The design of the coal drop system ensures that the material's exit velocity approximates the belt speed, thereby reducing induced wind speed and suppressing dust dispersion. With the advancement of discrete element method (DEM) simulation software and the enhancement of computer computing power, Cleary, Nordell, et al. employed DEM simulations to model material transport trajectories under actual working conditions. Their results demonstrated that designing the coal drop system in the transport system as a curve can more effectively control the flow direction of discrete materials and reduce the final material outlet velocity, thereby minimizing material impact and dust generation[6]. Building on the original cyclone dust collector design, H. J. Luckner [7] introduced additional modifications to enhance its efficiency for power plants. This improvement aimed to address the low dust removal efficiency despite mechanical dust removal technology's simplicity and ease of maintenance. Test results showed that the modified design expanded the range of captured particle sizes and significantly improved the overall dust removal efficiency of the collector. Xiang Xu[8] combined plasma with a ring electrode through capacitive coupling to refine the original electrode design and voltage levels, thereby enhancing dust removal efficiency. Experimental findings indicated that while dust removal efficiency increased, the required dust removal time increased for larger dust particles. Heinz Antes[9] introduced circulating electrostatic dust removal technology into the mine belt transportation system to reduce dust diffusion in large spaces. Experimental results demonstrated that this technology can achieve a dust reduction efficiency of up to 90% in the workplace. However, it also involves high power consumption and significant manual maintenance requirements, increasing operational costs. Shi Jinhua et al.[10] also applied spray sprinkler technology to control dust in the belt coal conveying system, achieving satisfactory dust suppression effects. Due to climatic limitations, the dust removal equipment and pipelines often freeze during cold winters, causing water conveyance and spray systems to malfunction. Zhang Wenbin et al.[11] proposed using bag filter dust removal methods to reduce dust concentrations to meet relevant national standard. Nevertheless, this approach suffers from the short service life of the cloth bags, necessitating timely replacement after accumulating a certain amount of coal dust, thereby increasing the maintenance workload. Moreover, inadequate or damaged bag sealing can increase coal dust pollution. Some scholars[12] have suggested employing micro-power dust removal technology for dust control. However, this method also incurs high power consumption and requires substantial manual maintenance, contributing to elevated operational costs.

In summary, the current domestic and foreign scholars ' research on dust removal technology mainly focuses on the technical improvement of the original dust removal device and the comprehensive application of the existing dust removal technology. Given the characteristics of the working conditions of the transshipment point of the coal conveying system, aiming at the problem of excessive dust control at the material transshipment point, the author takes Huaibei Coal Preparation Plant in Anhui Province of China as the research object. Based on the dust generation mechanism analysis and migration law, the idea of applying the curved coal drop pipe combined with the unpowered dust removal device to comprehensively control the problem of excessive dust at the transshipment point is proposed.

(6) **Why was the standard k-ε turbulence closure model was used the simulations? Did the authors try different turbulence models?**

**Reply：**In the process of numerical simulation and analysis, we also consider some other turbulence models used in the past for comparative analysis. However, in the process of using different models (such as the k-ω model, LES model, etc. ) for calculation, it is found that the calculation convergence effect is not ideal. Of course, it may not be all the problems of the model itself. Based on the research content of others ' literature and the broad applicability of the k-ε model, the k-ε turbulence closure model was finally selected for numerical simulation.

**In article：**

**4 Gas-solid two-phase flow theory calculation equation**

The blanking process at the coal transfer point involves a gas-solid two-phase flow, where air is the continuous phase. Given that the volume fraction of coal particles does not exceed one-third of the total flow field volume, the discrete phase model (DPM) is appropriate for the calculations. The power generated by the airflow during the unloading process primarily originates from the induced airflow caused by the coal particles, necessitating a thorough consideration of the coupling effect between the gas and solid phases in the simulation. Additionally, particle collisions during the blanking process significantly influence the movement of coal particles and the distribution of the flow field, thus requiring the inclusion of a particle collision model. In this study, the Discrete Element Method (DEM) collision model was selected for analysis. Since the steady state of the airflow field within the dust collector is a transient process, the model is solved using unsteady-state conditions. Leveraging the high precision and broad applicability of the standard k-ε turbulence model and considering the specific characteristics of the blanking process, the standard k-ε turbulence model was chosen for the simulation.

(7) **The authors need to clarify how they examined if the flow was fully developed.**

**Reply：**In response to the reviewer's comments, we have clarified our methodology for examining whether the flow was fully developed.

**In article：**

**5 The influence of key technical parameters on the dust removal efficiency of dust collector**

We measured the wind speed and pressure distribution at different cross-section positions along the airflow direction. By analyzing the stability of the velocity profile along the wind speed flow direction and the pressure drop characteristics along the flow direction, we can determine whether the internal flow of the dust collector has been fully developed. Based on the full development of the internal flow of the unpowered dust removal system, we carried out a numerical simulation analysis on the relevant physical parameters affecting the dust removal efficiency. The specific research results are as follows.

(8) **Can authors justify the inclusion of computational mesh in detail? However, the mesh dependency studies are not very convincing. The authors may consider using one of the more sophisticated methods, such as the Grid Convergence Index (GCI), which is commonly applied and recommended by the Fluids Engineering Division of the American Society of Mechanical Engineers as a reliable method for mesh independence analysis. This methodology is described in P. J. Roache, "Perspective: a method for uniform reporting of grid refinement studies," 1994 and applied in much research.**

**Reply：**We are very grateful for the valuable learning materials provided by the reviewer. We know that the quality of grid division will significantly impact the accuracy and stability of the calculation results. As far as grid division technology is concerned, we will further strengthen learning in the future. In this study, we use the hexahedral meshing method. When considering the quality of meshing, on the one hand, we are based on some essential criteria for the quality of meshing (such as Skewness, Aspect Ratio, Jacobian Ratio, etc.). On the other hand, we try to compare the accuracy of the calculation under different grid sizes by changing the minimum size of the grid unit and observing the data of the measuring points.

**In article：**

In the computational grid division of the physical model, an unstructured grid division algorithm is utilized to discretize the domain with varying cell sizes. Cell sizes range from 60 mm to 120 mm in increments of 10 mm. Mesh quality is rigorously evaluated using established criteria such as Skewness, Element Quality, Aspect Ratio, Warping Factor, and Jacobian Ratio, et al. Wind speed and wind pressure monitoring points are strategically placed within the model to examine the impact of different grid sizes on simulation accuracy. This enables the acquisition of pressure and wind speed curves at each monitoring point. The results indicate that when the grid size is less than 100 mm, the convergence of wind speed and wind pressure calculation results from each monitoring point is satisfactory, with deviations in monitoring data across different grid sizes controlled within 5%. Therefore, considering both the accuracy of the calculation results and the efficient use of computing resources, this study ultimately adopted a grid size of 100 mm as the standard. The final grid division outcome is presented in Figure 4.

(9) **Can authors justify the inclusion of boundary conditions in detail?**

**Reply：**Thank you for the insightful questions raised by the review expert. In the initial modeling stage, according to the relevant references and engineering practice, we have developed some basic boundary conditions for the model. In future studies, we will focus on justifying the inclusion of boundary conditions mentioned by reviewers.

**In article：**

**3.2 Parameter setting**

According to the fundamental principle of turbulence in numerical simulation, and considering the specific circumstances of coal transfer points, the model parameters were established[16] as presented in Table 1.

**Table 1 Model parameter setting**

| **Parameter** | **Parameter setting** | **Parameter** | **Parameter setting** |
| --- | --- | --- | --- |
| Solver | Pressure basis solver | Entrance and exit pressure | 0 Pa |
| Turbulent flow model | Standard k-ε model | Turbulent intensity | 4% |
| Entrance boundary type | Pressure inlet | Solution method | SIMPLEC |
| Exit boundary type | Pressure outlet | Convergence criteria | 1e-06 |

(10) **Additional details on the reliability and significance of the validation test used to evaluate the differences between experimental and numerical simulation values should be included.**

**Reply：**Thank you for your question, the operation law of the materials in the coal pipe at the transfer point of the coal conveying system was numerically simulated. An in-depth analysis of the dust generation mechanism and its causes in the belt coal conveying system was conducted, leading to the optimization of process parameters for the unpowered dust removal system. The research findings have been successfully implemented in the retrofit project of the existing belt coal conveying system at the Huaibei Coal Preparation Plant. This implementation effectively curtails dust generation at its source, significantly reduces dust concentration in the working environment, and ensures employees' occupational health and safety. To further validate the accuracy of the simulation results, a similar experimental simulation platform will be constructed for additional verification, as illustrated in Figure 2.

**Fig.1 Test platform and measuring point layout**

**In article：**

**6.2 Dust sampling test results**

The coal handling system's belt conveyor at the plant has been in a state of disrepair for an extended period, leading to misalignment with current production capacity. Furthermore, the inherent limitations of specific specialized mechanical equipment have rendered the existing dust removal system incapable of meeting the required production environmental standards. It is proposed that a transformation be undertaken based on the operational principles of unpowered dust removal technology and parameter optimization simulation results. Through comprehensive on-site investigations and analyses of multiple belt conveyor transfer points, the belt coal conveying systems in typical scenarios, such as the raw coal unit, washing unit, and transfer station within the coal preparation plant's conveying unit, were selected for pilot transformation. Sampling and testing were conducted at each location. By comparing and analyzing the test results, the practical application effects of the unpowered dust removal system utilizing reflux pipes were evaluated (the physical device on site is shown in Fig.10).

……

**6.3 Dust control effect analysis**

……

In summary, transforming the unpowered dust removal system in the coal preparation plant has substantially mitigated the issue of dust levels exceeding standards at each transfer point. Post-transformation, the dust concentration at all transshipment points was reduced to below the occupational exposure limits for workplace dust, fully complying with national and international regulations. This not only effectively safeguards the health and safety of workers but also significantly reduces the risk of occupational pneumoconiosis.

(11) **The critical discussion part has to be enlarged. You need strong discussion in your study. It has to be improved to pin-point the actual novelty(s) in this study. The results are only presented, but a proper discussion is missing.**

**Reply：**According to the reviewer’s comment, the critical discussion part has been enlarged.

**In article：**

**5.1 Analysis of the influence of return pipe on dust removal efficiency of dust collector**

……

From the analysis of the velocity cloud diagram, it is evident that during the accelerated falling process of the coal flow, the disturbance in the nearby flow field generates a high-speed induced airflow within the blanking tube. When the material flow strikes the bottom belt, a high-speed impact airflow is produced, and at the bottom of the guide groove, high-speed swirls are generated due to wall blockage. At this point, the wind speed at the outlet of the guide groove can reach 1.05 m/s. According to the pressure cloud diagram analysis, the positive pressure at the junction of the coal conveying belt and the coal drop pipe reaches its peak, leading to a sharp increase in air pressure at the blanking point. Due to the limited release space, the air volume induced by the primary dusting effect also reaches its maximum. Upon collision with the belt, the coal flow generates a strong air shock wave, causing the dust-laden air mass to spread evenly around the blanking pipe, resulting in severe dust dispersion. This phenomenon makes it easy for dust and powder to escape from this area. Simultaneously, the surrounding air is disturbed again and induced to form a secondary airflow under the pressure exerted by the subsequent coal flow. The secondary airflow combines with other airflows, diffusing the dust-laden air mass into the surrounding environment. The dust source fills the entire working space through repeated, continuous action.

To control dust generation at its source, a non-powered dust removal technology is proposed to transform transfer points in existing belt coal conveying systems based on coal dust's generation mechanism and diffusion characteristics. This technology primarily employs aerodynamics and pressure equilibrium principles, utilizing a closed-loop circulation balance device (reflux pipe) to control material flow. By balancing the induced wind pressure inside the guide groove with the external space pressure, this method effectively mitigates the high-pressure environment and high-speed induced airflow and impact airflow generated during the blanking process, thus preventing dust spillage caused by poor sealing of the guide groove. The key advantage of this technology lies in its ability to significantly reduce the velocity of the gas-solid two-phase medium without any external power, thereby inhibiting dust generation during the material falling process. Numerical simulations have been conducted to obtain the velocity and pressure contours of the airflow field under this working condition, as illustrated in Fig. 6.

……

The discrete element method (DEM) model is utilized to simulate the movement of coal flow particles, with the trajectory of material particles illustrated in Fig. 7. As shown in the diagram, after dust enters the reflux tube under the influence of the drainage plate, its kinetic energy decreases due to particle collisions. Following collisions with the tube wall, the particles adhere to the surface and gradually coalesce into larger particle clusters. Guided by airflow and gravity, these particles settle naturally. By comparing dust deposition and escape under two working conditions—with and without the return pipe (as shown in Table 4)—the results demonstrate that the return pipe's installation significantly enhances the device's dust removal efficiency by 24.3%.

**5.2 Analysis of the influence of the diameter of the return pipe on the dust removal effect of the dust collector**

**……**

Based on the characterization parameters of the dust removal efficiency of the dust collector, an increased pressure difference across the return pipe leads to more significant fluid energy loss. When the return pipe diameter is 500 mm, the pressure difference peaks, resulting in a pronounced decompression and deceleration effect within the dust collector. As the velocity of the dust decreases during internal migration, it becomes more likely for the particles to settle or coalesce into larger aggregates, eventually falling onto the surface of the conveyor belt.

**5.3 Analysis of the influence of the horizontal installation position of the return pipe on the dust removal effect of the precipitator**

**……**

According to the characterization parameters of the dust removal effect, when the horizontal distance is 2000 mm, the pressure difference across the return pipe reaches its maximum, resulting in the highest fluid energy loss and the most significant pressure and velocity reduction by the dust collector. At this distance, the material can descend along the coal pipe wall at a regulated speed to minimize dust generation and reduce airflow velocity. This approach, combined with dust deposition treatment at the blanking point, overcomes the limitations of traditional dust removal methods. The dust control mechanism is primarily reflected in the following aspects: the negative pressure airflow generated during the material's descent is channeled through the return pipe, causing the induced airflow to linger within the inertial coal drop pipe and increasing the collision frequency of coal dust particles. Consequently, the kinetic energy of the coal dust particles is converted into potential energy, reducing their velocity and allowing them to gradually settle onto the coal conveyor belt, thereby confining the dust production process to the flow within the coal drop pipe.

Lastly, we extend our sincere gratitude for the exceptional and scholarly revision of our manuscript.
