# Supplementary material for: Numerical simulation study on optimization of key technical parameters of unpowered dust removal system in a gas-solid two-phase flow field
Source: PLoS One. 2025 May 30;20(5):e0324379. doi: 10.1371/journal.pone.0324379 (PMC12124849; doi:10.1371/journal.pone.0324379)
Supplement: Attachment — Submitted filename: Response_to_the_reviewers_auresp_2.docx [file pone.0324379.s003.docx]

**Response to the reviewers**

Dear Prof. Muhammad Shakaib,

Thank you very much for your letter and the comments from the referees about our paper submitted to ***Plos One.***

We have checked the manuscript and revised it according to the comments. We submit here the revised manuscript as well as a list of changes.

If you have any question about this paper, please don’t hesitate to let me know.

Sincerely yours,

Lv Chen

**Response to Reviewer 1:**

Thanks for your comments on our paper. We have revised our paper according to your comments:

**(1) The numerical simulation is conducted with the Ansys Fluent software. The specificic version of the software should also be declared.**

**Reply：**According to the reviewer’s comment, We have provided the specific version of the software.

**In article：**

**3.1 Physical model and meshing**

The equal-proportion simplified physical model was established using SolidWorks modeling software, as illustrated in Fig. 3. Subsequently, this model was imported into Ansys-Fluent 2023R1 numerical simulation software to perform the subsequent simulation calculations.

**(2) Most of the references cited in the text are used in the introduction. In Section 4 dedicated to explain the continuous and discrete phase only reference [15] is cited and that reference is published in Chinese in the Journal of China Coal Society.**

**In the numerical methodology some decisions taken should be justified according to the documentation of the program and other relevant publications in the literature.**

**Reply：**According to the reviewer’s comment. In the section 4 of this study, we present a comprehensive review of relevant published literature on gas-solid coupling and other numerical simulation methods.

**In article：**

**4 Gas-solid two-phase flow theory calculation equation**

The blanking process at the coal transfer point is characterized as a gas-solid two-phase flow, with air acting as the continuous phase. Given that the volume fraction of coal particles does not exceed one-third of the total flow field volume, the Discrete Phase Model (DPM) is deemed suitable for computation. The airflow field power generated during the unloading process at the transfer point primarily stems from the induced airflow caused by coal particle movement. Therefore, the coupling effect between the gas and solid phases must be carefully considered in the simulation. Additionally, during the blanking process, collisions between coal particles can significantly influence the coal particles' motion and the flow field's distribution. Consequently, it is crucial to incorporate the particle collision model into the analysis. This study investigates the phenomenon by activating the DEM Collision Model option during the physical model setup. Since the airflow field within the dust collector reaches a steady state through a transient process, the model is solved using an unsteady-state approach. Considering the high precision and broad applicability of the standard k-ε turbulence model[16] and the specific characteristics of the blanking process, the standard k-ε turbulence model is selected for the simulation.

**4.1 Continuous phase analysis**

The Euler method is employed in this study to represent the motion parameters of the airflow field in the dust removal device. Given that heat transfer is not involved in the flow process, the fundamental physical principles encompass mass conservation and momentum laws. Neglecting variations in the density of the dusty airflow allows simplification to an incompressible viscous fluid motion problem. The mathematical representation of this control equation is as follows:

The continuity equation[17]:

$\frac{\partial\rho}{\partial t}+\frac{\partial}{\partial x_{i}}\left(\rho\mu_{i}\right)=0$ (1)

The momentum equation[18]:

$\frac{\partial}{\partial t}\left(\rho\mu_{i}\right)+\frac{\partial}{\partial x_{j}}\left(\rho\mu_{i}\mu_{j}\right)=-\frac{\partial p}{\partial x_{i}}+\frac{\partial\tau_{ij}}{\partial x_{j}}+\rho g_{i}-f_{i}$ (2)

In the formula, $\rho$ represents the fluid density, $x_{i}$ and $x_{j}$ denote the coordinates in the three-dimensional space ($x$,$y$and$z$), $\mu_{i}$ and $\mu_{j}$ represent the velocity components along each coordinate direction ($x$,$y$and$z$), $p$ signifies the fluid pressure, $\tau_{ij}$ denotes the stress tensor, $f_{i}$ represents the resistance of the fluid in each coordinate direction ($x$,$y$and$z$), while $g$ stands for gravitational acceleration.

**4.2 Discrete phase analysis**

Dust is subject to multiple forces during its migration and diffusion in the airflow field. The equation of motion for a single dust particle can be expressed as follows[15]:

$\sum F=m_{p}\frac{d\nu_{p}}{dt}$ (3)

In the formula, $\sum F$ represents the cumulative external force acting on an individual dust particle, where $m_{p}$ denotes the mass of the particle and $v_{p}$ signifies its velocity. The impact of forces on dust migration and diffusion in the flow field varies. Among these forces, pressure gradient force, additional mass force, and Basset force are considered negligible; however, gravity, buoyancy, aerodynamic drag, Saffman lift, and Magnus force significantly influence dust movement within a dust collector's flow field. Accurate characterization of particle behavior necessitates their precise calculation. During the migration of dust particles, collisions occur between particles and between particles and walls. The calculation equation for soft elastic collisions can be derived based on the methodology presented in Reference.[19].

**(3) In the abstract of the paper is declared that a soft collision model and that the Discrete Element Method is used but in the explanation of the discrete phase in section 4 details are not provided.**

**Reply：**According to the reviewer’s comment. We restructured the abstract and provided a more comprehensive foundation for the collision calculation in Section 4.

**In article：**

**Abstract:** Based on the engineering background of the Huaibei Coal Preparation Plant in Anhui Province, China, this study aims to effectively mitigate dust pollution during the blanking process at coal transfer points. Given the limitations of conventional dust control measures, a numerical simulation was conducted using the Euler model and discrete element method (DEM) for simulating particulate matter. The simulation incorporated the two-way coupling effect between gas-solid two-phase flow and the collision-adhesion dynamics among particles. We examined the migration behavior of dust within an unpowered dust removal system under various technical parameters. Results indicate that installing a return pipe significantly reduces dust overflow caused by impact airflow during blanking. Specifically, when the return pipe diameter is 500 mm, and the horizontal distance between the drainage port of the return pipe and the bottom of the blanking pipe is 2000 mm, the dust removal efficiency reaches its optimal level. Field tests confirmed that after implementing the improved dust removal system, the ambient air dust concentration decreased to less than 2 mg/m³, representing a reduction of approximately 92.02% compared to pre-transformation levels. This approach overcomes the limitations of traditional dust prevention technologies, inhibits dust generation at its source within the coal conveying system, effectively reduces working space dust concentrations, and ensures occupational health and safety for workers.

……

**4.2 Discrete phase analysis**

Dust is subject to multiple forces during its migration and diffusion in the airflow field. The equation of motion for a single dust particle can be expressed as follows[15]:

$\sum F=m_{p}\frac{d\nu_{p}}{dt}$ (3)

In the formula, $\sum F$ represents the cumulative external force acting on an individual dust particle, where $m_{p}$ denotes the mass of the particle and $v_{p}$ signifies its velocity. The impact of forces on dust migration and diffusion in the flow field varies. Among these forces, pressure gradient force, additional mass force, and Basset force are considered negligible; however, gravity, buoyancy, aerodynamic drag, Saffman lift, and Magnus force significantly influence dust movement within a dust collector's flow field. Accurate characterization of particle behavior necessitates their precise calculation. During the migration of dust particles, collisions occur between particles and between particles and walls. The calculation equation for soft elastic collisions can be derived based on the methodology presented in Reference.[19].

**(4) Ansys Fluent is a CFD solver package that uses the Finite Volume Method. It has the option to activate the Discrete Phase Model to model multiphase dispersed flows. In the Discrete Phade Model in recent versions the program incorporates the capability to model the interaction between particles using the DEM approach. In my opinion is very important to remark that diferences because there are other in-house and commercial solvers that are purely DEM.**

**Reply：**Thanks the reviewer’s comment. The recommendations provided by the evaluation experts were incorporated into the corresponding sections of the article with appropriate modifications to their content.

**In article：**

The blanking process at the coal transfer point is characterized as a gas-solid two-phase flow, with air acting as the continuous phase. Given that the volume fraction of coal particles does not exceed one-third of the total flow field volume, the Discrete Phase Model (DPM) is deemed suitable for computation. The airflow field power generated during the unloading process at the transfer point primarily stems from the induced airflow caused by coal particle movement. Therefore, the coupling effect between the gas and solid phases must be carefully considered in the simulation. Additionally, during the blanking process, collisions between coal particles can significantly influence the coal particles' motion and the flow field's distribution. Consequently, it is crucial to incorporate the particle collision model into the analysis. This study investigates the phenomenon by activating the DEM Collision Model option during the physical model setup. Since the airflow field within the dust collector reaches a steady state through a transient process, the model is solved using an unsteady-state approach. Considering the high precision and broad applicability of the standard k-ε turbulence model[16] and the specific characteristics of the blanking process, the standard k-ε turbulence model is selected for the simulation.

**(5) I do not understand some sentences in the introduction "In 1986, American scholar Martin Engineering [5] developed Inertial Flow Technology (IFT) ". Martin Engineerng is a company.**

**Reply：**Thank you for the insightful questions raised by the review experts. We have reorganized the content of the introduction and thoroughly examined this type of issue.

**In article：**

**0 Introduction**

**……**

Since the 1960s, numerous domestic and international scholars have initiated research on dust control technology. As awareness of the hazards associated with dust has increased, significant advancements have been made in this field. Various innovative dust removal equipment, technologies, and materials have been continuously introduced[1-4]. In 1986, American Martin Engineering Company[5] developed Inertial Flow Technology (IFT). This technology focuses on designing a novel curved coal drop system. Controlling the trajectory of falling material to align closely with the inner wall of the coal drop system forms a stable material flow, thereby reducing collisions between the material and the inner wall and among material particles, thus minimizing dust generation. The design of the coal drop system ensures that the material's exit velocity approximates the belt speed, thereby reducing induced wind speed and suppressing dust dispersion. With the advancement of discrete element method (DEM) simulation software and the enhancement of computer computing power, Cleary, Nordell, et al. employed DEM simulations to model material transport trajectories under actual working conditions. Their results demonstrated that designing the coal drop system in the transport system as a curve can more effectively control the flow direction of discrete materials and reduce the final material outlet velocity, thereby minimizing material impact and dust generation[6]. Building on the original cyclone dust collector design, H. J. Luckner [7] introduced additional modifications to enhance its efficiency for power plants. This improvement aimed to address the low dust removal efficiency despite mechanical dust removal technology's simplicity and ease of maintenance. Test results showed that the modified design expanded the range of captured particle sizes and significantly improved the overall dust removal efficiency of the collector. Xiang Xu[8] combined plasma with a ring electrode through capacitive coupling to refine the original electrode design and voltage levels, thereby enhancing dust removal efficiency. Experimental findings indicated that while dust removal efficiency increased, the required dust removal time increased for larger dust particles. Heinz Antes[9] introduced circulating electrostatic dust removal technology into the mine belt transportation system to reduce dust diffusion in large spaces. Experimental results demonstrated that this technology can achieve a dust reduction efficiency of up to 90% in the workplace. However, it also involves high power consumption and significant manual maintenance requirements, increasing operational costs. Shi Jinhua et al.[10] also applied spray sprinkler technology to control dust in the belt coal conveying system, achieving satisfactory dust suppression effects. Due to climatic limitations, the dust removal equipment and pipelines often freeze during cold winters, causing water conveyance and spray systems to malfunction. Zhang Wenbin et al.[11] proposed using bag filter dust removal methods to reduce dust concentrations to meet relevant national standard. Nevertheless, this approach suffers from the short service life of the cloth bags, necessitating timely replacement after accumulating a certain amount of coal dust, thereby increasing the maintenance workload. Moreover, inadequate or damaged bag sealing can increase coal dust pollution. Some scholars[12] have suggested employing micro-power dust removal technology for dust control. However, this method also incurs high power consumption and requires substantial manual maintenance, contributing to elevated operational costs.

……

**(6) I should explain in more the transition from the real geometry to the computational domain used in the simulations. And in that model I would explain in more detail the boundary conditions used. In Figure 3 it can be observed the presence of a dust curtain and a dust filter but when the numerical methodology is presented nothing is said about how they are modeled.**

**Reply：**Thanks to the valuable questions raised by the review experts, the dust curtain in the model was configured based on the porous medium theory. Specifically, the viscous resistance coefficient was set to 5×e^7 m⁻², the inertial resistance coefficient was set to 80 m⁻¹, and the downstream surface of the dust curtain was designated as a "Trap."

**(7) An unsteady simulation is performed but nothing is said about the temporal scheme and the time step used. All the results are presented without making any mention about the time.**

**Reply：**Thanks to the valuable questions raised by the review experts, the setting parameter of time step is supplemented in this paper.

**In article：**

**Table 1 Model parameter setting**

| **Parameter** | **Parameter setting** | **Parameter** | **Parameter setting** |
| --- | --- | --- | --- |
| Solver | Pressure basis solver | Entrance and exit pressure | 0 Pa |
| Turbulent flow model | Standard k-ε model | Turbulent intensity | 4% |
| Entrance boundary type | Pressure inlet | Solution method | SIMPLEC |
| Exit boundary type | Pressure outlet | Convergence criteria | 1e-06 |
| Wall function | Non-Equilibrium Wall Functions | Time step size | 0.001 s |

**(8) Nothing is said about the treatment of wall-bounded turbulent flows. Are you using standard wall functions or a enhanced wall treatment? Does the mesh used satisfied the requirements of yplus values?**

**Reply：**Thanks to the valuable questions raised by the review experts, “Non-Equilibrium Wall Functions” are used. The mesh used satisfied the requirements of y+ values, the height of the first layer grid is 0.15 mm, and the grid growth rate is 1.2.

**In article：**

**Table 1 Model parameter setting**

| **Parameter** | **Parameter setting** | **Parameter** | **Parameter setting** |
| --- | --- | --- | --- |
| Solver | Pressure basis solver | Entrance and exit pressure | 0 Pa |
| Turbulent flow model | Standard k-ε model | Turbulent intensity | 4% |
| Entrance boundary type | Pressure inlet | Solution method | SIMPLEC |
| Exit boundary type | Pressure outlet | Convergence criteria | 1e-06 |
| Wall function | Non-Equilibrium Wall Functions | Time step size | 0.001 s |

**(9) In my opinion the subsection 6.2 should be eliminated from the article. A numerical simulation study should only be validated with results published in the literature**

**Reply：**We extend our gratitude to the experts for their valuable suggestions. After careful deliberation, we have decided to retain the content of Section 6.2. The purpose of retaining this section is not to validate the accuracy of the simulation results but to evaluate the actual dust removal performance of the unpowered dust collector under the current research parameter settings.

**Lastly, we extend our sincere gratitude for the exceptional and scholarly revision of our manuscript.**
